# Factors that influence evidence-informed meso-level regional primary health care planning: a qualitative examination and conceptual framework

**DOI:** 10.1186/s12961-023-01049-8

**Published:** 2023-09-25

**Authors:** Alice Windle, Sara Javanparast, Toby Freeman, Fran Baum

**Affiliations:** 1 College of Nursing and Health Sciences, Flinders University, Adelaide, SA Australia; 2 Stretton Health Equity, Stretton Institute, The University of Adelaide, Adelaide, SA Australia

**Keywords:** Evidence-informed, Health planning, Policy-making, Primary health care, Framework, Regional

## Abstract

**Background:**

Evidence-informed primary health care (PHC) planning in decentralised, meso-level regional organisations has received little research attention. In this paper we examine the factors that influence planning within this environment, and present a conceptual framework.

**Methods:**

We employed mixed methods: case studies of five Australian Primary Health Networks (PHNs), involving 29 primary interviews and secondary analysis of 38 prior interviews; and analysis of planning documents from all 31 PHNs. The analysis was informed by a WHO framework of evidence-informed policy-making, and institutional theory.

**Results:**

Influential actors included federal and state/territory governments, Local Health Networks, Aboriginal Community Controlled Health Organisations, local councils, public hospitals, community health services, and providers of allied health, mental health and aged care services. The federal government was most influential, constraining PHNs’ planning scope, time and funding. Other external factors included: the health service landscape; local socio-demographic and geographic characteristics; (neoliberal) ideology; interests and politics; national policy settings and reforms; and system reorganisation. Internal factors included: organisational structure; culture, values and ideology; various capacity factors; planning processes; transition history; and experience. The additional regional layer of context adds to the complexity of planning.

**Conclusions:**

Like national health policy-making, meso-level PHC planning occurs in a complex environment, but with additional regional factors and influences. We have developed a conceptual framework of the meso-level PHC planning environment, which can be employed by similar regional organisations to elucidate influential factors, and develop strategies and tools to promote transparent, evidence-informed PHC planning for better health outcomes.

**Supplementary Information:**

The online version contains supplementary material available at 10.1186/s12961-023-01049-8.

## Background

Health policy formulation by national and state governments is complex, as is much clinical practice decision-making by service providers. It is well documented that such decisions are not always informed by evidence. In between these two realms of macro-level decisions on policy initiatives, and micro-level clinical decisions, sits the meso-level of regional primary health care (PHC) planning. Meso-level, primary health care (PHC) organisations are responsible for assessing local population health needs, and planning primary health care services and population health programs. They have defined geographical boundaries and can cover metropolitan or rural regions, or a combination. Primary health care (PHC) organisations are well placed to identify and respond to local health needs, involve and empower local communities in decision-making and facilitate integration of local services. Several high-income countries have PHC organisations as part of their healthcare systems: Primary Health Organisations in New Zealand; a variety of structures in different provinces in Canada; and Clinical Commissioning Groups (CCGs) in the UK [1]. In low and middle income countries similar roles are played by District Health Systems [2]. In Australia, there have been three iterations of PHC organisations that have been implemented in local areas, covering the entire country: 31 Primary Health Networks (PHNs) (since 2015) that replaced Medicare Locals (MLs) (from 2011 to 2015) that in turn replaced Divisions of General Practice (from the early 1990s to 2011). The exact structures, functions, and governance of PHC organisations differ somewhat. However, they all serve as a layer of regional PHC planning and decision-making that is devolved from and funded by the federal government. PHC organisations are regional, ‘above’ organisations with individual PHC practitioners. Australia’s latest iteration, Primary Health Networks (PHNs), are tasked with planning and commissioning services and programs to address identified local needs. This largely entails clinical healthcare services (medical and allied health), behavioural health promotion programs, and local health system integration. PHNs contribute to public health in a broad sense and operate in a complex environment where public health and healthcare responsibilities are split among federal, state and local governments [3].

An important principle of primary health care (PHC) is that it is ‘scientifically sound’ – that decisions are informed by robust, reliable evidence [4]. Health policy and clinical practice interventions that are not informed by evidence risk being ineffective, inappropriate, wasteful and potentially harmful. So too do meso-level PHC planning decisions, yet this level of health planning and decision-making has received scant research attention. To promote the use of evidence to inform health decision-making, it is essential to understand the context or environment in which policy or planning takes place, and how it is influenced [5]. This paper aims to provide such understanding regarding meso-level PHC planning.

Primary Health Networks have some autonomy to address local population health needs and responsibility for allocating considerable public funds. As such, this meso-level of PHC decision-making warrants deeper understanding and critical analysis of the factors that influence planning and the use of evidence. This research examines the factors that influence evidence-informed planning in PHNs, as a contemporary example of meso-level PHC organisations, and proposes a conceptual framework representing a range of influential factors.

While there is some published academic literature on interventions implemented by PHC organisations [6], there is relatively little about the organisations’ processes and functions. Several studies have specifically examined the use of evidence for regional planning by English CCGs [7–9], but do not extend to the broader contextual factors that influence decision-making. Borek et al. [10] examined the social and contextual influences on antimicrobial prescribing in CCGs. Several studies have examined evidence-informed decision-making in Australian local government [11–13], but no such examination of PHNs’ (or their predecessors’) evidence-informed planning has occurred.

PHNs operate in a highly political context and understanding the broader ‘policy system’ or environment in which they operate is important in examining their planning and decision-making. Our earlier related research [3, 14–16] has provided important findings regarding the broader context and drivers within which planning decisions are made. Australian PHC organisations have been shown to have limited collaboration with local government [14] and state /territory government actors [3]. PHNs’ predecessors (MLs) were strongly influenced by the regulatory institutional forces and biomedically focussed ideas of health of their government funders. This conflicted with the values and normative forces within the organisations and constrained their upstream health promotion actions [15]. The pursuit of comprehensive PHC in Australian PHC organisations has been hindered by the interests and power of the medical sector driving a clinical focus, as well as neoliberal ideas of economic imperatives and market models [16]. While these studies provide a rich understanding of the broad external contextual factors influencing PHNs, analysis of the more proximal and internal influences is lacking. Efforts to implement evidence-informed innovation, and to improve the use of evidence to inform planning require contextual knowledge. Such ‘mapping’ of the meso-level PHC organisation planning ‘environment’ in the form of a conceptual framework, is absent in the literature.

Lessons from health policy-making are valuable in examining health planning decision-making at the meso-level. Contemporary accounts of policy-making recognise the dynamic and iterative ‘messiness’ and complexity, and advocate understanding of a policy ‘system’ or ‘environment’ [17–19].

**Fig. 1:**
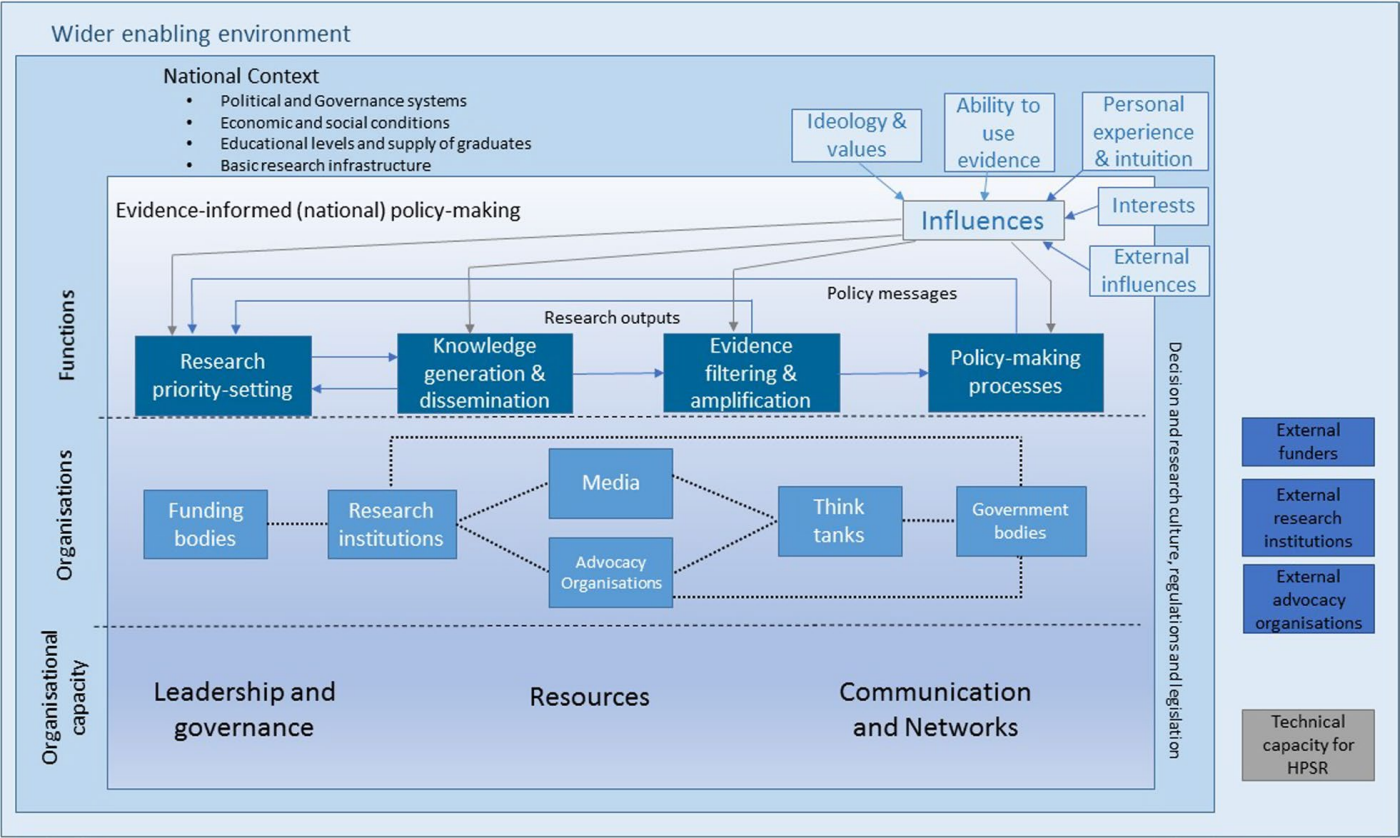
WHO Conceptual Framework for Evidence-Informed Health Policy-Making (reproduced from Green and Bennett, 2007.). Reprinted with permission of the World Health Organization, from Sound choices: enhancing capacity for evidence-informed health policy, Green, A. & Bennett, S, Page 51, Copyright 2007. Available at [https://apps.who.int/iris/bitstream/handle/10665/43744/9789241595902_eng.pdf?sequence=1], accessed 29 June 2021

There are many theories, models and frameworks of evidence-informed health decision-making. Milat and Li [20] identified 41 frameworks for translating research into policy and practice. The field of implementation science abounds with frameworks to support the implementation of evidence-based innovation in clinical practice. These frameworks have been categorised as determinant, process and/or evaluation, depending on what purpose they serve [21]. Green and Bennett [5], under the auspices of the World Health Organization, developed a conceptual framework for evidence-informed national policy-making (Fig. 1) (hereafter referred to as the ‘WHO Framework’ for brevity). The WHO Framework takes a ‘systems thinking’ approach to delineate the multiple factors and elements that comprise the policy environment.

Given the scarce research attention to the meso-level PHC organisation planning environment, and the lack of a conceptual framework, this paper had two aims:

- To present findings from a study using the WHO Framework as the basis for an examination of the factors and actors that influence evidence-informed health planning in Australian PHNs;
- Based on this study, to present a conceptual framework designed to improve understanding of the planning environment of meso-level PHC organisations

## Methods

Study methods are reported according to COREQ criteria. See Additional file 1.

### Research context and methods overview

This paper reports research on the PHN planning environment. The study builds on a large mixed-methods research program that ran from 2014 to 2018, funded by the Australian National Health and Medical Research Council (APP#1064194). The larger study examined various aspects of Australian PHNs and MLs: comprehensive PHC approaches, population health planning, corporate governance, health equity and more. This PhD sub-study was initiated by the PhD candidate (first author) who had previously worked in a (non-participating) PHN and ML. The research was overseen by a Critical Reference Group which included representatives from participating PHNs, state and federal government, and other key stakeholders.

This research employed various methods. The principal approach was case studies of five PHNs drawing on primary interviews (conducted in 2018), plus secondary analysis of interview data obtained in 2016 as part of the overarching project. All interview data analysis examined contextual elements of the PHC planning environment, and planning processes, and the 2018 interviews also examined organisational capacity for evidence-informed planning. Analysis of all 31 PHNs’ public planning documents also contributed to this research, complementing the focussed case study analysis with a broader examination of the entire range of PHNs.

### Data collection

#### Case studies

##### Case PHN recruitment

A purposive sample of PHNs were recruited. Initially, the six PHNs who had participated as cases in the earlier stages of the research were invited to continue. An email invitation outlining the proposed PhD research project was sent to the PHN Chief Executive Officers (CEOs) by chief investigator and principal supervisor (second author). Two PHNs declined, with one citing lack of capacity for staff to participate in research, and the other not willing to take part in the research. Four agreed to continue. Two other PHNs with a similar geographic profile to those who had declined were identified and invited, of which one accepted. The final sample comprised five PHNs, from five of Australia’s eight states/territories. Two were in metropolitan areas, one in a rural area, and two included both metropolitan and rural areas. To ensure anonymity, participating PHNs are referred to as Metro North, Metro South, Rural North, Rural South, and Remote. The CEO of each PHN gave consent for the organisation to participate.

##### Secondary analysis of 2016 interviews

The methods for recruiting participants and conducting the 2016 interviews are described elsewhere [14]. The breakdown by PHN of the 38 2016 interviews that were used for secondary analysis is shown in Table 1. These interviews were conducted with members of boards, Clinical Councils, Community Advisory Committees, as well as PHN senior executives, managers and staff. While the objectives and focus of this later PhD component of the research were different, the potential for relevant information from the main project indicated the value of conducting secondary analysis of 2016 interview data. Much relevant background information about the settings, influences, stakeholders and activities of the PHNs had already been obtained. The secondary analysis focussed on examining contextual factors, influences and actors in PHNs’ planning environment. Additional file 2 details where 2016 interview data were used to address some of the research questions.

**Table 1:** PHN interview participant numbers

PHN (codename)	Number of 2016 interviews	Number of 2018 interviews
Metro North	10	6
Metro South	7	6
Rural South	11	6
Remote	10	6
Rural North	Not applicable	5

##### 2018 Interviews

Participating PHNs were invited to nominate six interview participants, representing the board, Clinical Council and Community Advisory Committee, CEO (or deputy), a senior manager and a staff member involved in planning and program development. One PHN nominated only five participants because they only had a small team to draw from. One of the invited interviewees declined, and a replacement was nominated. All interviewees gave informed consent to participate in the research, and none withdrew.

Of the 29 interviews, 20 were conducted face-to-face at the respective PHN, and nine via telephone. Interviews were conducted in private and ranged in duration from approximately 60 to 80 min. Interviews took place between May and September 2018, and none were repeated. Interviews were semi-structured, guided by an interview schedule developed by the research team, to address the research questions, and informed by the theoretical framework, including the elements of the WHO framework (See Additional file 2). Two pilot interviews were conducted with people from non-participating PHNs. We refined the interview schedule following each of the pilot interviews, adjusting the order of questions, adding prompts for further probing questions in order to elicit appropriate detail and refining the wording and clarity of questions. We also created a separate version of the interview schedule for board, clinical council and community advisory committee interviewees. This version excluded the majority of the questions about organisational capacity as the pilot interviews showed they required knowledge of operational detail which was not reasonable to expect from members of the PHNs’ governance structures. Interviews were conducted by the first author, a PhD candidate with experience in qualitative interviewing, and in PHC organisation planning roles. Two of the interviewees had prior professional peer interactions with the interviewer, and the rest had only a preliminary introduction to the research and interviewer prior to participation. All interviews were digitally recorded and all but one were professionally transcribed. Due to poor sound quality, one interview was transcribed by the interviewer. Field notes were made during and after each interview.

#### Document analysis

The publicly available needs assessments, activity work plans and annual reports of all 31 PHNs were analysed, to examine factors influencing PHC planning. Documents were analysed using NVivo, with data coded according to a framework developed by the research team, to address the research questions. Document analysis was conducted prior to, and supplemented interview analysis in identifying factors that influence evidence-informed planning. Its primary purpose in the broader research project was to examine the extent and types of evidence used to inform planning (reported elsewhere [22]).

### Theoretical approach

The coding framework and analysis in this research was informed by several theoretical and conceptual frameworks. The WHO Conceptual Framework for Evidence-informed Health Policy-making [5] provided a ‘systems thinking’ approach to unpack the complex environment of PHC planning in PHNs. The research also draws on Makkar et al.'s [23] ORACLe Tool domains of organisational capacity for evidence-informed health policy-making to examine such factors in PHNs.

An institutional theory ‘lens’ was employed, to consider the underlying role of actors, ideas and institutional forces on the phenomena in the PHN planning environment. Institutions are social structures that have a high degree of resilience, whose stability and meaning is provided by underlying normative, regulative and cultural-cognitive forces [24]. *Normative* forces relate to values, ideas and social ‘norms’ of how things should be, *regulative* forces relate to the rules and obligations of how things are required to be, and *cultural-cognitive* forces relate to actors’ worldview and conceptions of the reality of how things are [24].

Underpinning this research with a strong theoretical basis aids in generalising the findings to different contexts, nationally and internationally. Detailed reporting of methods and results also aids the transferability of the research, such that readers can make informed judgements on whether the findings could be reasonably transferred to other settings [25].

### Coding and analysis

Interview data were analysed using NVivo qualitative analysis software [26] and coded according to a framework [27] informed by the theoretical approach outlined above. The framework initially used to analyse 2016 interview data did not directly inform the coding framework for this later phase of the research, other than both employing an institutional theory lens [24]. Two interviews from different PHNs underwent ‘familiarisation’ analysis [27] using the draft coding framework, and additional codes were added inductively [28]. The coding framework was refined through discussion among the research team.

Transcripts from 2016 interviews were coded prior to conducting the 2018 interviews, to provide background information.

Checked and cleaned 2018 interview transcripts were read through in detail, with text coded to relevant nodes. A deductive coding approach was employed, grouping data where it appeared to align with any of the pre-selected, theoretically-informed codes.

The first author coded all transcripts and inter-coder reliability testing was carried out on nine transcripts by the other 3 authors. There was generally strong agreement between coders, with some minor discrepancies between similar nodes.

Data from 2016 and 2018 interviews were thematically analysed together noting the differences between PHNs. Thematic analysis is a method for identifying, analysing and reporting patterns or themes within data. A theme captures something important about the data in relation to the research question, which represents some degree of patterned response or meaning within the data set [29, 30]. Emerging issues were noted and sub-themes inductively developed within the main themes/codes. Any divergent views were also noted.

### Member checking

Several strategies were employed to check participants’ intended meaning [25]. All interviewees were offered the opportunity to review their transcript prior to analysis. Research findings were communicated to all participants, in the form of a summary, policy brief and the complete thesis. Participants were offered further detailed description and/or discussion of individual case findings, however none opted to.

### Reflexivity

It is important to acknowledge that the research was led, and interviews were conducted by a PhD candidate (first author) who had worked in PHC organisations. She was aware of the influence that this experience had on the research as she brought a good deal of background contextual knowledge. The researcher was an outsider to the case study organisations, but an insider in terms of having previously worked in similar organisations.

In interviews, the researcher probed to ensure she understood what the interviewee meant, and avoided assumptions. She took field notes following each interview to prompt reflection on her previous experiences in PHC organisations and whether there were points that she agreed or disagreed with in the interview.

The researcher made reflexivity ‘disclosure’ notes in the margins of drafts, to indicate where aspects of the research had reminded her of something from her experience. These were discussed in research team meetings about data interpretation and emerging findings. The researcher also consciously looked for, and reported dissenting views in the data, to ensure that she wasn’t just focussing on what she agreed with, or what resonated with her. These reflexivity approaches ensured that the experience that the researcher brought to this study enhanced understanding of the interview data and drew on her experience without it unduly colouring the interpretation.

## Findings

First we focus on our findings about the powerful influence of the federal government on PHNs. Next we present the other actors and factors that we identified as influencing PHNs’ PHC planning. We will then present a conceptual framework for the PHC organisation planning environment, developed based on these findings.

### The powerful influence of the federal government

Our research identified the federal government Department of Health—the funder of PHNs—as having the strongest influence on PHNs’ planning. The federal government tightly regulates PHNs, limiting the funding and timeframes for planning, and constraining the scope of activities PHNs can plan and commission. This theme was frequently identified, by 28 of 29 (97%) 2018 interviewees, and 33 of 38 (87%) 2016 interviewees in all PHNs. Interviewees’ comments highlighted the strength of this restrictive influence, for example:

*“We are still however in a fairly authoritarian environment where a lot of it is pre-set for us.” (Senior Executive, Rural South, 2018)*

*“the fact is that the funding streams, by and large, are tied very closely to specific objectives of the government.” (Clinical Council, Metro South, 2018)*

*“it’s becoming more and more micro-managed…I think it’s lost its ability to make as big a difference as it could, because of the lack of choice of how to spend the money and what to do” (Clinical Council, Rural South, 2018)*

PHNs’ remit was seen to be limited to a selective conception of primary care, which emphasised medical services. This reflected the federal government policy direction, political appetite and neoliberal, individualistic ideas of health which aligned more with service delivery than system reform (first quote below) or prevention through action on the social determinants of health (second quote below).

*“one of the things I think that frustrates us is a drive by the department for service delivery compared with system reform… we probably believe it's wiser for us to invest a small amount of resources in system reform, which will then hopefully change a system that will have a long-lasting impact rather than just putting our finger in the dyke by just funding gaps in the service delivery landscape year by year.” (Manager, Metro North, 2018)*

*“I think there’s a very good understanding around the table of the place of social determinants of health, … but in terms of the job we’re being given to do at the moment, it is more focused on health services and the clinical side of health services, there isn’t must room for prevention yet, there isn’t much room for social determinants.” (Board, Metro South, 2016)*

The limitations to PHNs’ planning scope reflected the individualistic ideology of the prevailing conservative government and a selective, clinically-focussed interpretation of PHC, which act as a strong cultural-cognitive institutional force:

*“unfortunately we’re in a ‘blue’ [conservative party] phase at the moment, and the ‘blue’ phase is not overtly [sic] friendly to an equity based approach to health service delivery. They just want the veneer of everyone getting the same” (Senior Executive, Rural South, 2018)*

*“It was an obvious shift in the political landscape with the change of government, where Medicare Locals and AMLA [MLs’ peak body] were talking a lot about issues about equity and inequality and social disadvantage and social determinants of health. A lot of that discussions seemed to have been shut down quite abruptly by the change of government. It came back to the equal distribution of the technical aspects of health care rather than the broader discussion about the determinants of health.” (Board, Remote, 2016)*

Several interviewees felt that the influence of federal government often outweighed evidence in determining priorities:

*“as much as we might like to think we’re totally objective in our decision making and priority setting, often we are forced to go down a path that is less attractive or less logical or less evidenced, simply because the political risks of not doing so in an environment where government wields a very heavy influence over the outcomes and activities of PHNs” (Senior Executive, Rural South, 2018)*

Many interviewees were concerned about PHNs’ limited funding. This issue was mentioned by 10 of 29 (34%) 2018 interviewees, and 20 of 38 (53%) 2016 interviewees. As found in earlier phases of the research [15], inadequate ‘flexible funding’ for commissioning interventions to address local priority issues was a prominent concern. In our present study, several interviewees identified the tension between PHNs’ requirement to comprehensively assess local needs, but their very limited funding to address identified priorities.

*“it’s a bit silly in fact because we do this whole [needs assessment] process and only end up with a couple of million dollars to actually then commission, that is actually flexible.” (Senior Executive, Metro North, 2016)*

*“the PHN has a mandate to do a comprehensive health care needs assessment. Great. They go and do that, but if the funding conditions that are set out by the Commonwealth [federal government] don't support meeting those needs, it puts them in a really awkward position … They know what the issues are but actually they then don't have the flexibility to use the funds in a way that will enable meeting what those community needs and priorities are.” (Community Advisory Committee, Remote, 2016)*

The present study further identified that PHNs’ operating budget was also underfunded, which hindered their capacity for fundamental functions that constitute robust, evidence-informed planning and commissioning such as: stakeholder and community engagement; partnerships; needs assessment and planning (and the evidence resources to inform that); contract management; and research and evaluation.

*“We operate within the environment where we’re given around 8% to operate on* …* It’s tiny, it is absolutely tiny… it’s not only lean, it’s gossamer thin and it’s inappropriate … you can expect leanness but there is a point that you go past where you say, “These organisations cannot possibly do the things that you want them to do in the way in which they should be done with that sort of amount of money to spend.”” (Senior Executive, Rural South, 2018)*

Limited funding (and flexibility) was seen to undermine the purpose of local needs assessment and planning.

Timeframes allowed by the Department of Health for PHNs to conduct needs assessment, planning and commissioning were concerning.

*“when we first were set up, it was sort of a mad rush, and we got set up in June, have a health needs assessment in November and your activity plans in by April.” (Manager, Metro North, 2018)*

Interviewees saw planning timeframes as “absurd”, “irrational” (Community Advisory Committee, Remote, 2018), “ridiculous” (Board, Metro South, 2018), “stupid” (Clinical Council, Rural South, 2018) and “unrealistic” (Senior Executive, Rural South, 2018). Short timeframes were seen to hinder evidence-informed planning in several ways. Rushed needs assessment meant compromising its depth and rigour, and therefore its value as evidence to inform decisions. Short deadlines meant that rushed planning hindered PHNs’ ability to investigate and develop appropriate, evidence-based interventions. We found vague, under-developed plans were submitted for (and received) departmental approval. Short planning timeframes also limited PHNs’ ability to engage meaningfully with communities and other stakeholders, to draw on locally specific, robust evidence. This was particularly an issue for engaging with First Nations people’s communities, which is vital to ensure the appropriateness, cultural safety and effectiveness of planned interventions.

### Other influential actors

Our research identified a wide variety of actors in the PHN planning environment. These included internal PHN staff, and a range of individuals and organisations associated through membership of PHNs’ Clinical Councils, Community Advisory Committees, Boards, other forums and informal networks. Interviewees indicated that some external actors had stronger influence than others, and this varied somewhat between PHNs. State/territory governments and Local Health Networks (LHNs) were perceived to have moderately strong influence. Local Health Networks (also known as Local Hospital Networks or Local Health Districts depending on the jurisdiction they are in) are state-based entities partially funded by the federal government [31]. They manage the delivery of public hospital services and other community-based health services across a defined geographical area as determined by the state/territory government [32]. The Department of Health [33] explicitly encourage strong engagement between PHNs and LHNs. Aboriginal Community Controlled Health Organisations and their peak bodies had strong engagement and influence in some PHNs, and less in others. Interviewees reported other influential organisations as local councils/government, public hospitals, allied health providers, community health services, mental health service providers and aged care providers. Individuals perceived to have strong influence were politicians and to a lesser extent, general practitioners, although GP engagement varied between PHNs. While many other actors (including community members and organisations) were identified by interviewees, they were not specifically identified as having strong influence on planning. Additional file 3 lists individuals and organisations from different sectors who are involved in relationships with PHNs, indicating their degree of influence, as identified by interviewees.

### Factors that influence PHN planning

As well as the strong influence of the federal government, we identified a wide range of factors that influence PHNs’ planning, and use of evidence. These stem from within the organisations, and from the regional and wider external contexts.

The influential factors are detailed in Additional file 4. External factors included: the health service landscape; local socio-demographic and geographic characteristics; (neoliberal) ideology; interests, power and politics; broader national policy settings and reforms, and federalism; and PHC reorganisation. Internal factors included: organisational structure; culture, values and ideology; various capacity factors; planning processes; transition history; and personal and professional experience.

### Development of the framework

We found the WHO Framework [5] (developed for national policy-making environments) did not entirely capture the broad range of factors and actors that influence meso-level regional PHC planning. We sought to develop an expanded PHC organisation environment conceptual framework, drawing on our findings, to map influential elements in the meso-level health planning environment. Additional file 4 shows how each of the identified factors informs and is situated on the framework, according to the level of context in which the factors are evident.

### PHC organisation environment conceptual framework

**Fig. 2:**
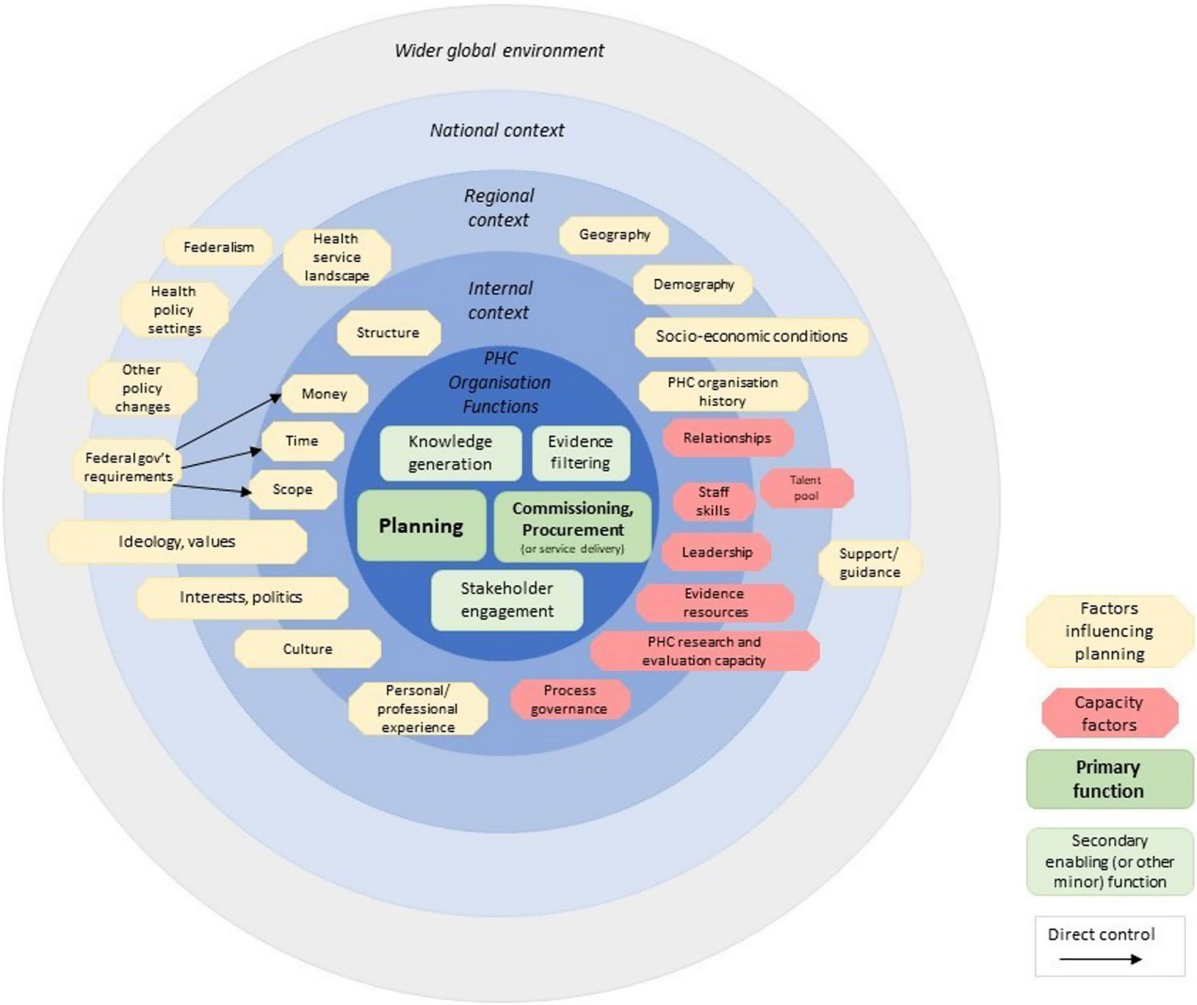
PHC organisation environment conceptual framework

Our conceptual framework of evidence-informed, meso-level PHC planning is shown in Fig. 2. This framework provides an overview of the meso-level PHC planning environment, and illustrates that it is complex, involving multiple functions and influenced by many contextual factors and influences, among which evidence is just one.

Our framework puts the functions of PHC organisations at the centre, and represents in concentric circles the influences and the contextual levels from which they derive.

Planning and commissioning/procurement are represented as the primary functions of PHNs. As well as planning for ‘flexible funding’ activities, PHNs develop plans for particular focus areas such as mental health, alcohol and other drugs, and First Nations peoples’ health. PHNs devote considerable effort and resources to ‘knowledge generation’ including needs assessment research, stakeholder engagement activities, pilot projects and evaluation. Stakeholder engagement is represented as a distinct function in the framework, yet our research identified that this also contributes to the other functions of ‘knowledge generation’, ‘planning’ and ‘commissioning and procurement’. Our framework also notes ‘direct service delivery’ within the commissioning/ procurement functions, which occurs in select circumstances in some PHNs. While the functions are represented in the framework as discrete ‘boxes’ there is considerable overlap and iteration between functions – they do not occur in isolation or sequentially. Our findings show that (evidence-informed) planning is but one function for PHNs, among many other tasks and priorities.

Our framework includes several capacity factors, relating to both individuals and organisations, that act at the internal, regional and national levels. It represents our findings that capacity factors at regional and national levels can influence capacity at more proximal context levels. For example, limited capacity for PHC research and evaluation in the region hinders such capacity within the organisation.

We identified considerable variation between regional factors in different PHNs, indicating the importance of local engagement and planning for effectively responding to local issues. Responsiveness to local context and priorities is at the core of PHC organisations’ purpose as devolved health planning organisations [34], and is a key principle of comprehensive PHC [4].

Our identification of the influence of neoliberal ideas and interests in regional PHC planning, led us to including an outer circle of the framework to represent these broader, international contextual influences.

Some factors span the levels of context, for example socioeconomic conditions have influence in all contexts.

The concentric circular arrangement of our framework represents the respective influence of distal factors in the outer contexts on more proximal factors in the inner contexts. At the inner level of the framework, regional factors influence internal issues, for example, health workforce shortages in rural and remote PHNs are a key contextual constraint on planning. At the outer level, external contextual factors have both direct and indirect influence on inner factors. For example, the federal government is responsible for national health policy which indirectly influences PHN planning. Our interviewees identified that the federal government also directly limits the scope of PHC interventions that PHNs develop. This reflects a strong regulatory influence in the external context, ultimately dominating over internal culture. This position of the PHNs illustrates a somewhat unique problem for meso-level health planning organisations. There is a fundamental tension in their position – they have some autonomy and a responsibility to identify and respond to local issues and priorities, yet their autonomy is considerably constrained, to the extent that their local responsiveness is hindered. They are in a tug of war between the local priorities and federal government regulatory forces. We also identified that some factors that act at multiple context levels are not consistent. For example there was a contest of ideas between some PHNs who wish to act on the social determinants of health, and the neoliberal federal government policy which favoured individualistic, bio-medical approaches.

Our examination of the factors that influence PHNs’ evidence-informed planning identified a broad range of considerations at varying levels of context. This framework serves to illustrate the complexity of that planning environment.

## Discussion

This section will highlight the contributions made by our framework, discuss its potential to drive evidence-informed PHC planning, and present some limitations of the research.

### What the PHC organisation environment conceptual framework adds

Our framework is the first to describe the ways in which the ‘policy environment’ of PHC organisations is influenced by regional and national forces. Many frameworks concerned with evidence-to-policy/practice are process frameworks that seek to guide the translation or transfer of knowledge into policy or practice. Our framework differs from such process frameworks in that it is a ‘determinants framework’ which broadly illustrates the constructs or factors (determinants) that directly and indirectly influence meso-level PHC planning. It breaks down the context (or environment) into its constituent parts. Determinant frameworks provide an important foundation for building more specific theories of change, in this case promoting evidence-informed PHC planning.

The WHO Framework, which informed our analytical framework, distinguished between the national context and the wider enabling environment. Our research approached the analysis with the simple distinction of external and internal context, as proposed by Dobrow et al. [35]. They make the important distinction that internal context factors can be modified by the organisation, but external factors are out of their control. Our analysis has identified that external context factors can be national or regional, which is a key feature of our framework and an important finding from our research. Understanding the context in which influences occur can assist organisations in adapting to or managing influences to improve evidence-informed decision-making [36].

Our framework features a concentric circular arrangement reflecting the different levels of context, and the source and inter-relationship of influences. This illustrates how PHC organisations’ decision-making is as complex as national policy-making, and fraught by tension due to an additional ‘layer’ of regional influences.

Our framework distinguishes between individual, organisational and regional/national capacity factors that impact on evidence-informed meso-level PHC planning.

The broader (international) environment is represented in our framework, to reflect the influence of forces that traverse national borders, such as neoliberalism. The rise of neoliberalism and its ideas of individualism, free markets and low regulatory constraints in healthcare, and bio-medical conceptions of health have contributed to the global failure to achieve comprehensive PHC [37, 38]. Our framework highlights the ways in which political and ideological factors affect planning. The influence of global and national neoliberal individualist, or socialist collective ideas of health indirectly impact on what types of evidence, for what purposes, are valued and considered (or not) in informing local policy and planning decisions.

Political and institutional factors can affect the use of health evidence including the framing of evidence in relation to social norms and values. The selection or interpretation of evidence used for health policy development has been shown to be biased by: values and moral convictions; religious and cultural identity; and nationalism [39]. Bacchi’s ‘problematization’ theory [40] describes how the way in which a problem is viewed will determine what strategies are adopted to address it, and that problematization is loaded with ‘ideas’ and value judgements underpinned by cultural-cognitive and normative institutional forces. This ideologically driven ‘problematization’ is a dominant regulatory institutional force from the federal government which constrains PHNs to clinical or individual solutions. As we report elsewhere [22], PHNs favour epidemiological and health services data as sources of evidence, which they are provided by the Department of Health. This is consistent with the government’s deeply held ‘ideas’ about health as a matter of disease (epidemiology) and its treatment (health service capacity and utilisation data), rooted in neoliberal, individualistic world views.

### Potential application of the framework

Our framework addresses a gap in the evidence-informed health decision-making literature. It also has potential practical application for Australian PHNs and PHC organisations and similar organisations in other countries that are responsible for devolved policy decision-making. The framework could also be used by such organisations’ respective ‘parent’ government agencies.

A determinants framework in implementation science can facilitate consideration of which factors can be influenced to improve healthcare implementation [41]. The WHO Framework can guide the assessment of countries’ existing capacities and contextual constraints to “enable the identification of key problems and the wise targeting of resources” for improving evidence-informed health decision-making ([2], p117). Likewise, our framework can be applied to guide the assessment of PHC organisations’ context, facilitating consideration of which factors can be modified (or not), to support initiatives to improve evidence use. It is less about implementation of innovations *by* PHC organisations to improve health—rather it concerns interventions or mechanisms *in* PHC organisations, to improve their evidence-informed PHC planning. Our framework can inform the improvement of evidence-informed planning processes to promote broad and transparent consideration of factors that influence planning decisions. While decision-making is highly dynamic and rarely a clear sequence, it can nonetheless benefit from a systematic and transparent process [42]. Decision-making should involve explicit description of the context and acknowledgement of influential factors [35], including the consideration and application of appropriate evidence [43]. Mechanisms that acknowledge the influences on decision-making represent a shift away from only building individuals’ capacity for evidence-informed decision-making. They represent a move towards what are increasingly recognised as more sustainable organisational and institutional capacity initiatives that embed and institutionalise governance, processes and resources [44]. As our framework illustrates, individual capacity is one factor among many that influence evidence-informed decision-making. Our framework can be used to inform planning tools that prompt the consideration of influential factors and promote transparent, evidence-informed decision-making. For example, informed by our framework, a PHC organisation may develop a template that prompts consideration and acknowledgement of factors that have influenced the selection/development of a given strategy, such as local influences, fit with national health policy and local socio-economic conditions. Such a template can then foster transparency as to why one strategy may have been selected over other options. Improved planning, incorporating greater understanding of the contextual factors that have influenced a planning decision, can help to ensure that planned strategies are designed and implemented effectively and appropriately. Well-designed and implemented strategies are more likely to achieve the intended population health outcomes.

Our framework can also aid the identification of aspects of the planning context that impede evidence-informed planning. Analogous healthcare implementation science frameworks [45] have informed the creation of instruments such as the Alberta Context Tool to assess aspects of the healthcare context, and identify aspects that are potentially modifiable [46]. Our framework, structured by context, clearly indicates which factors are in the internal/organisational context and readily modifiable by the PHC organisation, and which are in the regional or national context, and likely more fixed. Implementing change (in this case, increasing the use of evidence in planning) should be tailored to the context [47, 48].

### Study limitations and strengths

There are several limitations of this research as they relate to the development of this framework. Direct observation may have been better than interviews for examining planning processes, however was not feasible within the project’s capacity constraints, and such intense scrutiny may not have been welcomed by the PHNs. Nonetheless, this research did reliably indicate that PHNs lack systematic, transparent planning processes, and this makes for less effective evidence-informed planning. Our analysis of actors was limited to interviewees reporting actors’ level of influence. A social network analysis of the relationships and influences of the many actors evident in the PHNs’ planning environment may have provided more detail. However, for the purposes of our study broadly illustrating the involvement of key actors, our approach was sufficient.

We also note that documents analysed were those approved by the Department of Health. Examination of internal planning guidance documents (meta-policies) and un-approved drafts may substantiate interview indications of tensions between PHN planning intentions and Department restrictions.

Lastly, the framework was not discussed with interview participants, although it was included in the thesis which was circulated to them. The framework was discussed and refined among the research team, of which three had strong PHN research experience, plus the lead author with direct PHN and ML experience. We are confident that the research has resulted in an accurate representation of the PHC organisation planning environment.

## Conclusion

Our research has found that meso-level PHC organisation planning occurs in a complex environment of multiple factors that influence planning decisions and the use of evidence to inform them. From this, we have developed a conceptual framework. We argue that the PHC organisation environment is more complex than much national policy-making for two reasons. Firstly, the additional regional contextual factors. Secondly, the dominant, powerful influence of the federal government which constrains the scope of PHNs’ actions, and sets strict financial and time limits for planning and commissioning that hinder evidence-informed planning.

Regional PHC planning that identifies and responds to local issues and local stakeholders serves an important function in driving access and equity in PHC. Reliable, evidence-informed planning, is likely to result when actors understand the complexity of the planning environment and identify barriers and enablers to evidence use in decision-making. Our framework can be applied to enable assessment of the planning environment and context, and explicit acknowledgement of influential factors.

We recommend this framework be employed by PHC organisations (or other decentralised health planning/policy bodies), ‘parent’ government agencies, and research stakeholders. Together, they can use it to co-design and implement strategies to enhance planning processes and transparency, to promote evidence-informed regional PHC planning. Systematic, transparent, evidence-informed PHC planning will lead to effective and efficient strategies to equitably address local health priorities.

### Supplementary Information

**Additional file 1. **COREQ Checklist.

**Additional file 2. **Table linking research questions, interview questions, coding framework and underlying theory.

**Additional file3. **Table of actors in the PHN planning environment.

**Additional file4. **Detailed findings regarding factors influencing PHC planning.

## Data Availability

Interview data are not publicly available as participants did not give consent for this. The 91 PHN documents (needs assessments, activity work plans and annual reports) that were subject to document analysis are available upon request, and were publicly available from the respective PHNs’ websites at the time of data collection in 2017–18.

## Abbreviations

CCG: Clinical Commissioning Group
LHN: Local Health Network
ML: Medicare Local
PHC: Primary Health Care
PHN: Primary Health Network
