# Supplementary material for: Factors that influence evidence-informed meso-level regional primary health care planning: a qualitative examination and conceptual framework
Source: Health Res Policy Syst. 2023 Sep 25;21:99. doi: 10.1186/s12961-023-01049-8 (PMC10521552; doi:10.1186/s12961-023-01049-8)
Supplement: Supplementary file 2 — Additional file 2. Table linking research questions, interview questions, coding framework and underlying theory. [file 12961_2023_1049_MOESM2_ESM.docx]

**Additional File 2: Research questions, interview questions, coding framework and underlying theory**

Research questions focussed on in this paper:

- What does the PHN planning environment look like in terms of context, influences and actors?
- How do PHNs undertake the process of PHC planning and decision-making?
- Do PHNs have strong organisational capacity for evidence-informed planning? (This question is addressed in detail in Windle et al., 2021 (4), but is included here because capacity factors influence evidence-informed planning, capacity elements are included on the conceptual framework)

| **Research question** | **Interview questions (not necessarily in order asked)** | **Codes** | **Rationale/ theory/ literature considerations** |
| --- | --- | --- | --- |
| RQ1 What does the PHN planning environment look like in terms of context…? | IQ1.6 What sort of broader external influences or constraints does your PHN face with regard to what you can and can’t do?  (+ 2016 interview data) | Context   - External   - The federal Department of Health   - Scope limitation, inflexibility of funding     - Geographic scope/size   - Time constraints/pressures imposed externally | External context is a component of the WHO framework (1). Sub-codes were developed inductively |
|  | (2016 interview data) | Planning system/environment   - Corporate Governance structure/ mechanisms - PHN functions   - Research priority setting   - Knowledge generation/ dissemination   - Evidence filtering/amplification | These functions are elements of the WHO framework |
|  | IQ2.7 Can you give me an example that illustrates how the culture or leadership of your PHN supports or doesn’t support the use of evidence to inform planning? | Context   - Internal/organisational   - Culture/ values   - ML transition – simple or difficult? | ‘Ideology and values’ are an element of the WHO framework |
| RQ1 What does the PHN planning environment look like in terms of … actors? | IQ1.4 Who are the various stakeholders that have input into the planning, and how do they have input?  • Which stakeholders do you think have greater influence, who has less influence? Why is that? (Also draw on data from 2016 interviews) | Planning system/environment   - Actors, organisations | ‘Organisations’ is a section of the WHO framework |
| RQ1 What does the PHN planning environment look like in terms of … influences …? | IQ1.5 Can you think of any examples of particular interests/agendas of stakeholders that influence planning at your PHN?  •Which interests/agendas are most influential and why?  (+2016 interview data) | Influences on decision making   - Ideology/values - Ability to use evidence - Personal or professional experience and intuition - Interests/agendas (local political) - External influences   - Politics - Institutional influences (internal or external) | These influences are elements of the WHO framework  Institutional theory (2) – normative, cultural/cognitive or regulatory forces |
| RQ2 How do PHNs undertake the process of PHC planning and decision-making? | IQ1.1 Can you tell me about the primary health care health planning process in X PHN?  •How are priorities selected?  •How are planning decisions made? How do you arrive at what goes into your annual plan?  •Is there a defined, systematic process, or is it more ‘organic’?  •How are strategies/programs developed? | Planning system/environment   - Planning/ decision making process   - Ad hoc?   - Engagement with evidence | Policy-making processes is an element of the WHO framework, alongside other functions (listed above) |
| RQ4 Do PHNs have strong organisational capacity for evidence-informed planning? | Interview questions based on the ORACLe tool (3) – see our related paper (4) for details. | Organisational Capacity:   - Leadership*^#^ - Governance of planning* - Resources*   - Documented processes^#^   - Support and tools^#^   - Workforce capacity and staff training^#^   - Available evidence - Communication and networks* - Capacity for generating new evidence to support PHN work^#^ - Evaluation^#^ | Elements of the WHO framework (*)  based on ORACLe tool capacity domains (3) – see our related paper (4) for details (#) |

Key: *Elements of the WHO framework

^#^based on ORACLe tool capacity domains (3) – see our related paper (4) for details

1. Green A, Bennett S. Sound choices: enhancing capacity for evidence-informed health policy: Alliance for Health Policy and Systems Research, World Health Organization; 2007.

2. Scott WR. Institutions and Organizations: Ideas, interests, and identities: SAGE Publications; 2013.

3. Makkar SR, Turner T, Williamson A, Louviere J, Redman S, Haynes A, et al. The development of ORACLe: a measure of an organisation’s capacity to engage in evidence-informed health policy. Health Research Policy and Systems. 2016;14(1):4.

4. Windle A, Javanparast S, Freeman T, Baum F. Assessing organisational capacity for evidence-informed health policy and planning: an adaptation of the ORACLe tool for Australian primary health care organizations. Health Research Policy and Systems. 2021;19(1):1-11.
