# Supplementary material for: Factors that influence evidence-informed meso-level regional primary health care planning: a qualitative examination and conceptual framework
Source: Health Res Policy Syst. 2023 Sep 25;21:99. doi: 10.1186/s12961-023-01049-8 (PMC10521552; doi:10.1186/s12961-023-01049-8)
Supplement: Supplementary file 3 — Additional file3. Table of actors in the PHN planning environment. [file 12961_2023_1049_MOESM3_ESM.docx]

Additional File 4: Actors in the PHN planning environment

This table lists individuals and organisations from different sectors who have involvement and relationships with PHNs, as identified from interviewees. This does not include PHN employees but does include members of boards and councils/committees. The degree of influence is indicated, with a high number of stars (*) indicating strong influence, (as perceived by interviewees) and no stars indicating that these actors were not noted as having significant influence on planning, merely identified by interviewees as having input.

Actors in the PHN planning environment

| **Sector** | **Organisations** | **Individuals** |
| --- | --- | --- |
| Government | - Federal government*** - State/territory government** - Local Health Networks** - Local councils/government* | - Politicians**, including Ministers* - Bureaucrats - Policy-makers, planners |
| Health service providers | - General practices** (Private, corporate) - Hospitals – public* - Hospitals – private - Emergency departments - Ambulance - Pharmacy - Allied health providers* - Aboriginal Community Controlled Health Organisations/ Aboriginal Medical Services** - Women’s health - Family violence - Community health services* - Charities, non-government organisations - Telehealth providers - Mental health service providers* (adult/ youth/ child, public/ private) - Alcohol and other drug treatment | - General practitioners* - Public health physicians - Medical specialists - Geriatricians - Practise nurses - Midwives - Practice managers and admin staff - Psychologist - Physiotherapist - Podiatrists - Occupational therapists - Aboriginal Health Workers/ practitioners |
| Provider peak bodies, professional groups | - Royal Australian College of General Practitioners - Australian Medical Association - Aboriginal Medical Service/ ACCHO peak bodies** - Rural health workforce agencies - GP training providers - Pharmacy Guild - General practice groups (from former Divisions of General Practice) - Aged & Community Services Australia (aged care peak body) |  |
| Other service providers/ government departments | - Police - Housing/homelessness - Community services - Legal advisory service - Transport - Interpreters - Aged care* - Domiciliary care - Relationships Australia | - Social workers - Lawyers |
| Non-government organisations, charities | - Cancer Council - Australian Council of Social Services (branches) - Asthma Australia - Diabetes Australia (branches) - Kidney Health Australia - Heart Foundation - Stroke Foundation - CanTeen - Mental Health Australia - Council on the Ageing (COTA) - Neighbourhood Houses |  |
| Community, consumers | - Health consumers’ advocacy groups e.g. Consumers Health Forum - Ethnic Communities Council - Aboriginal elders/ community groups - Refugee advocacy groups - Carers Australia (branches) - Other community groups - Community advisory groups (associated with Local Health Networks) | - Community members - Carers - Refugees - Aboriginal and Torres Strait Islander people - People from culturally and linguistically diverse communities - Older people - LGBTIQ people - People with disabilities |
| Other | - Universities - Private health insurers - Australian Health Promotion Association - Australian Healthcare and Hospitals Association - Primary Care Partnerships - Other PHNs | - Academics/researchers - Accountants - Lawyers - Consultants |
