# Supplementary material for: Factors that influence evidence-informed meso-level regional primary health care planning: a qualitative examination and conceptual framework
Source: Health Res Policy Syst. 2023 Sep 25;21:99. doi: 10.1186/s12961-023-01049-8 (PMC10521552; doi:10.1186/s12961-023-01049-8)
Supplement: Supplementary file 4 — Additional file4. Detailed findings regarding factors influencing PHC planning. [file 12961_2023_1049_MOESM4_ESM.docx]

**Additional File 3: Detailed findings regarding factors influencing PHC planning**

| **Factor** | **Source of influence & position in framework** | **Key findings and quotes illustrating the influence** | **Implications for (evidence-informed) planning** |
| --- | --- | --- | --- |
| Evidence sources | Federal government – external actor influences PHN access to evidence resources. | The Department of Health indirectly influences which evidence informs needs assessment and planning, by providing access and directing PHNs to large national data sets via a PHN web portal, as the basis for their needs assessments. Evidence in these portals tends to be quantitative demographic, epidemiological and health services utilisation data. | Constrains evidence-informed planning. Drives PHNs towards bio-medically focussed, quantitative evidence sources |
| Inadequate support and guidance | Federal government – external actor that influences PHNs’ capacity and processes. | Interviewees strenuously criticised the lack of support or guidance by the federal Department of Health regarding planning and commissioning in the early stages of establishment, and the imposition of tight transition timeframes: *“I don’t think that there was really much help coming from them [the Department of Health]. In fact, I think they’re all scrambling desperately to work out how to do it all themselves.”* (Board, Metro South, 2016) | Impeded establishment of organisational capacity, robust planning processes and culture, and made for great uncertainty and stress at the outset. |
| Health service landscape | External context – local/regional. | The composition and distribution of healthcare services and workforce in the region influences local needs/priorities and planning options. This is particularly an issue in regional and remote areas where health workforce shortages are common. In service-rich areas integration between service providers took greater priority. | Workforce shortages limit options for planned services/programs to address local needs |
| Local geography | External context – local/regional. | Population density and remoteness vary among and within PHNs.  Geographies and local climate conditions can also influence population health needs. | Influences local health needs. Can constrain options for interventions to address needs. Impacts on service viability, distribution and access.  Distance/remoteness can limit stakeholder engagement and increase operational and program costs |
| Socio-demographic context | External context – local/regional | Different regions have diverse demographic profiles, with varying population proportions of Aboriginal and Torres Strait Islander people, people from culturally and linguistically diverse communities, refugees and migrants.  Some interviewees saw socio-economic factors to be important, with endemic poverty and social disadvantage reported as key issues in some regions. | The diversity of communities has implications for identifying and appropriately responding to community health needs, in collaboration with communities, which is integral to local health planning. Co-design is more time consuming, but more ethical, than top-down, non-participatory health planning.  Communities that face endemic hardship have many competing priority issues, many of whose underlying causes are beyond the scope of PHNs’ influence. Planned interventions need to be appropriate for communities with diverse needs and limited resources. |
| Interests, power and politics | Local health care service providers – actors in the local context. | Vested interests of local service providers in commissioning was a common theme. Some interviewees from Clinical Councils and Community Advisory Committees were quite explicit that their motive for becoming involved with the PHN was to advocate for their particular interest, such as allied health, community services or general practice. Interests were generally underpinned by altruistic motives for the greater community good.  Vested interests challenged objective, evidence-informed decision-making when PHNs were redistributing or decommissioning services:  *“it doesn’t make the role of a PHN as a commissioner easier when you’ve got this self-interested influence coming in at multiple levels that threatens to separate you from the objectivity of your planning and data analysis process that’s evidencing where you need to invest” (Senior Executive, Rural South, 2018)*  The influence of the federal government’s ideas of health reflects the broader contextual influence of neoliberal ideology, with associated conservative, bio-medical, individualistic ideas and approaches, and the indirect influence of this global phenomenon on local planning. | Interests challenge or outweigh evidence in determining local priorities and activities.  Constrains scope of planned interventions |
| Interests, power and politics | Politicians – actors in the national external context. | Interviewees identified various influences from politicians: their preferences for certain types of services, high profile ‘ribbon cutting’ opportunities; and interest in particular issues or advocacy on behalf of local lobby groups.  *“ministers like cutting ribbons to services… in a highly urbanised context like this, a lot of what we could do is make a difference around systems and processes and referral pathways; but ministers don't like that as much. They like ‘here's the service’ with the doctor standing there with their stethoscope.” (Senior Executive, Metro North, 2018)* | Influence PHN priorities and planning, or circumvent planning processes (also indicative of ideological influence of politicians, with bio-medical focus) |
| Broader policy settings and federalism | Factors in the national external context. | The federated, dual funding of health system(s) in Australia was frequently noted as an factor influencing PHC planning (see (1) also)  The (Medicare) fee-for-service based model of primary care funding was recognised as an immutable contextual challenge.  *“I don’t think fee-for-service really encourages the primary care then to take the time that is necessary for these [chronic disease] patients, and the most developed countries have gone for a hybrid model away from fee-for-service.” (Clinical Council, Remote, 2016)*  Interviewees identified the ‘freeze’ on Medicare rebates as a contextual factor influencing planning. It has impacted GP finances and viability, and driven increases in patient co-contribution for GP services.  *“because of the intensity of the issues such as the MBS freeze … it is difficult for GPs to be able to step up above their current challenges to their practices and to their profession and really to take that population health approach.” (Clinical Council, Rural South 2016*) | Adds to complexity of planning. Potential for cost and responsibility shifting  Planned interventions need to work within the scope of Medicare or have enough funding to be sustainable without Medicare. Also drives and reinforces bio-medical ideas/conceptions of health  Environment of constrained finances hinders capacity for change and innovation in primary care sector |
| Other sectors’ major policy reforms | Factors in the national external context | Significant changes in other related sectors such as disability and aged care were noted as contributing to a dynamic environment.  “*I do think that there has been so much reform and so much change, and it’s happening across sectors, like NDIS [National Disability Insurance Scheme], aged care, within health, within general practice, that it’s about how you get your voice heard within such a dynamic environment. I think that’s also challenging.” (Manager, Rural South, 2018*) | Dynamic policy environment. Change fatigue among PHN and external stakeholders |
| Defunding of major national PHC research institutions | Capacity factor in the national external context | The Primary Health Care Research and Information Service (PHCRIS), and Australian Primary Health Care Research Institute (APHCRI) were recently discontinued. Federal government funding was withdrawn. | Curtailed national capacity for generating evidence to inform PHC planning. Hinders availability of quality Australian PHC evidence. |
| History of frequent reorganisation of PHCOs prior to PHNs. | Factor in the national external context | Sixty-one Medicare Locals were established to replace Divisions of General Practice between 2011 and 2013, yet the incoming federal government disestablished them in 2015, and replaced them with 31 PHNs. PHNs were selected via a competitive tender process, which was widely criticised for its timing and lack of transparency (2).  Perceived vulnerability of PHNs to political whim, and concerns about longevity following the highly political demise of MLs:  *“the concern I’ve got is how many iterations [of commissioning cycles] are we going to get?” (Board, Metro South, 2018).* | Change fatigue among PHN and external stakeholders. Reorganisation and mergers in some PHNs hindered establishment of planning processes and disrupted stakeholder relationships.  Uncertainty about organisational longevity has implications for stakeholder engagement, job security and staff retention, which can impact on capacity for evidence-informed planning |
| Transition history | A factor in the internal and local context | There were varying experiences and outcomes of the transition from MLs to PHNs. Some PHNs enjoyed a relatively simple and smooth transition from one ML to one PHN:  *“I think we’ve had a very stable organisation and I think capacity and experience was maintained” (Manager, Metro North 2016)*  For others the transition was very disruptive, with mergers or takeovers of 2-3 organisations, and significant changes to leadership, staffing, funding and activity.  *“We were starting pretty much from scratch in terms of setting up policies, procedures, systems, all that kind of thing” (Board, Metro South, 2018)*  The rushed transition reportedly disrupted stakeholder engagement. | PHNs that had a relatively straightforward transition benefitted from established systems, whereas those who had more complicated transitions were at a disadvantage in having to re-establish organisational systems, processes and cultural norms to facilitate robust evidence-informed PHC planning.  Stakeholder engagement is critical to locally relevant PHC planning. Without adequate engagement, planned strategies may not be appropriate for the community. |
| Organisational structure | A factor in the internal context | Organisational structures vary somewhat between PHNs, in terms of organisational hierarchies, and some examples of more than one Clinical Council or Community Advisory Committee, plus other bodies. | Differing arrangements had implications for evidence-informed planning processes and stakeholder influence. |
| Capacity for evidence-informed planning  (PHC research and evaluation capacity, evidence resources, process governance, talent pool, relationships, leadership, staff skills) | Various factors in the internal, regional and national contexts | Organisational capacity for evidence-informed planning was relatively strong overall, however varied across different ‘domains’ of capacity, and between organisations. (see 3)  In summary, PHNs had strong capacity for generating evidence and in relationships with researchers. There was weak capacity regarding evaluation processes, staff training, evidence resources and guiding tools to support evidence-informed planning. Leadership capacity to drive evidence-informed planning varied.  We also identified individual factors, such as analysis skills, ability to source and appraise evidence, and established relationships that contribute to the capacity for evidence-informed planning within an organisation.  Rural PHNs reported difficulty recruiting appropriately skilled and experienced planning staff. | Weak capacity hinders evidence-informed planning, and strong capacity enables it.  The capacity of individuals within an organisation can greatly influence evidence-informed planning.  A smaller pool of ‘talent’ within the region indirectly constrains PHNs’ planning capacity |
| Planning processes | A factor in the internal context, with some external influence by the Department of Health | Interview data regarding planning processes tended to lack clarity and detail, suggesting that relevant organisational norms were not yet firmly established.  There were mixed views about rigour in PHN planning processes and where they sat on a spectrum of ‘disorganised’ to ‘systematic’. While some were confident in their PHNs’ planning processes, others recognised that there was room for improvement, particularly in the more systematic, direct sourcing and use of evidence for specific planning purposes, rather than sporadic and indirect use.  As reported elsewhere (3), we identified some shortcomings in PHNs’ capacity regarding governance of the planning process, with limited mechanisms for promoting the appropriateness, transparency, accountability and contestability of evidence use. | Evidence-informed decision-making can be hindered by a lack of systematic planning processes with mechanisms to promote the use of evidence. |
| Unexpected priority changes, shifting ‘goal posts’ | A factor in the external context | Many interviewees noted that PHNs required flexibility and responsiveness to shifting “goal posts” (Community Advisory Committee, Remote, 2016), particularly for responding to political influences.  *“I think in this field you have to be, especially in [region], flexible with your planning because much can change on a whim, especially within the political environment.” (Community Advisory Committee, Remote, 2018)* | The need to respond to sudden changes can divert PHNs’ focus, compromise the strategy of planning processes, and impact their capacity, performance and outcomes. |
| Culture, values, ideology | Factors in the internal PHN context | Our interviewees indicated that the culture and values within PHNs broadly favour evidence-informed decision-making, although some noted the contest between good intentions and time constraints.  Our analysis of the organisational values that PHNs’ stated in their public documents found that ‘evidence’ was rare, with only 3 of 29 PHN organisations stating this as a value. The most frequently stated values were respect (n=18), collaboration (n=18) and innovation (n=16). | Where evidence is valued and its use is a cultural ‘norm’, this enables evidence-informed planning |
|  | Tension between internal and external values and ideology | Some interviewees expressed ideas consistent with a social model of health, valued action on the social determinants of health, and were frustrated that the federal government limited their activities to individualistic approaches centred on clinical services and behaviour change strategies, consistent with neoliberal ideology. | This reflects a tension between strong regulatory forces driving the ideology of federal government influences, and weaker internal PHN ideological and cultural influences.  As outlined above, ideologically constrained scope limits options for effectively and equitably addressing the root causes of health needs and gaps. |
| Personal and professional experience | Factor in the regional external and internal PHN context | Influential experience from actors included: ‘on the ground’ experience of local clinicians; lived experience of service users and the wider community; staff and other actors’ previous experience in MLs and DGPs; research and academic expertise among staff and advisory groups; and population health experience.  Some were concerned that the predominance of clinical experience narrowed ideas of health. Others recognised the importance of balancing clinical and population health experience and ideas to help broaden the scope of PHN actions:  *“bringing together a composition of clinical as well as community representatives and population health - I think that’s a good thing. I think it increases the scope of what we can do, it increases our ability to implement wider and broader strategies that are a bit more inclusive of things like the social determinants of health. Rather than just looking at primarily downstream and reductionist models of health.” (Staff, Metro South, 2016)* | Experience can help to contextualise evidence for the local setting, and ensure that planned activities are appropriate and acceptable. |

1. Freeman T, Baum F, Javanparast S, Ziersch A, Mackean T, Windle A. Challenges facing primary health care in federated government systems: Implementation of Primary Health Networks in Australian states and territories. Health Policy. 2021;125:495-503.
2. Thompson, J. The costly abolition of Medicare Locals. Australian Policy Online. 2015. <https://apo.org.au/node/58917> (Accessed 12 May 2017)
3. Windle, A, Javanparast, S, Freeman, T, Baum, F. Assessing organisational capacity for evidence-informed health policy and planning: an adaptation of the ORACLe tool for Australian primary health care organizations. Health Research Policy and Systems. 19(1):1-11
